# BUILDING A STRATEGIC PARTNERSHIP: TURNING CHALLENGES INTO OPPORTUNITIES

**DOI:** 10.1093/geroni/igad104.1483

**Published:** 2023-12-21

**Authors:** Pamela Elfenbein

**Affiliations:** University of North Georgia, Oakwood, Georgia, United States

## Abstract

The University of North Georgia’s Institute for Healthy Aging (IHA) and the Georgia Department of Human Services, Division of Aging Services (DAS), have partnered to address critical workforce needs in the program areas of Elder Abuse, Neglect and Exploitation and Public Guardianship. Working together they have aligned requirements a new University System of Georgia degree, the Nexus degree, with the educational and experiential requirements of employment in these DAS program areas. The University System of Georgia’s Nexus Degree, a new academic credential and the first new degree program in the United States since the 1890s when the associate’s degree was added, was designed to help more Georgians access careers in high demand areas. Creation of this degree is in direct response to talent demand analysis with employers in high demand career areas. The Division of Aging Services faces many challenges that hamper finding and retaining the right workers for the difficult but rewarding roles in Adult Protective Services and Public Guardianship. This presentation will outline the DAS needs and UNG academic program, and how working together to meet the needs of at-risk elders, a direct pipeline to employment has been developed. This paper will discuss and provide insight into this unique collaboration and newly created training approach.

